# MicroRNA-211 Expression Promotes Colorectal Cancer Cell Growth *In Vitro* and *In Vivo* by Targeting Tumor Suppressor CHD5

**DOI:** 10.1371/journal.pone.0029750

**Published:** 2012-01-03

**Authors:** Chunxiao Cai, Hassan Ashktorab, Xiaowu Pang, Yuan Zhao, Wei Sha, Yulan Liu, Xinbin Gu

**Affiliations:** 1 Department of Gastroenterology, Peking University People's Hospital, Beijing, People's Republic of China; 2 Department of Oral Pathology, Howard University, Washington, D.C., United States of America; 3 Department of Medicine, Howard University, Washington, D.C., United States of America; 4 Cancer Center, Howard University, Washington, D.C., United States of America; University of Barcelona, Spain

## Abstract

**Background:**

Chromodomain-helicase-DNA-binding protein 5 (CHD5) is a newly identified tumor suppressor that is frequently downregulated in a variety of human cancers. Our previous work revealed that the low expression of CHD5 in colorectal cancer is correlated with CHD5 promoter CpG island hypermethylation. In this study, we investigated the effect of microRNA-211 (miR-211)-regulated CHD5 expression on colorectal tumorigenesis.

**Methodology/Principal Findings:**

miR-211 was predicted to target CHD5 by TargetScan software analysis. A stably expressing exogenous miR-211 colorectal cancer cell line (HCT-116^miR-211^) was generated using lentiviral transduction and used as a model for *in vitro* and *in vivo* studies. The expression level of miR-211 in HCT-116^miR-211^ cells was upregulated by 16-fold compared to vector control cells (HCT-116^vector^). Exogenous miR-211 directly binds to the 3′-untranslated region (3′-UTR) of CHD5 mRNA, resulting in a 50% decrease in CHD5 protein level in HCT-116^miR-211^ cells. The levels of cell proliferation, tumor growth, and cell migration of HCT-116^miR-211^ cells were significantly higher than HCT-116^vector^ cells under both *in vitro* and *in vivo* conditions, as determined using the methods of MTT, colony formation, flow cytometry, scratch assay, and tumor xenografts, respectively. In addition, we found that enforced expression of miR-211 in HCT-116 cells was able to alter p53 pathway-associated regulatory proteins, such as MDM2, Bcl-2, Bcl-xL, and Bax.

**Conclusion/Significance:**

Our results demonstrate that CHD5 is a direct target of miR-211 regulation. Enforced expression of miR-211 promotes tumor cell growth at least in part by downregulating the expression level of the CHD5 tumor suppressor. Our results provide a better understanding of the association of between miR-211-regulated CHD5 expression and CHD5 function in colorectal tumorigenesis.

## Introduction

Identifying cancer-related genes and understanding their contribution to tumorigenesis are critical steps in controlling cancer. Recent studies have demonstrated that gene expression can be affected by changes in chromatin structure and the association of DNA with nucleosomes [1]. For example, Swi/Snf proteins can cause ATP-dependent disruption of nucleosome structure at a promoter, which enhances the binding of transcription factors to their binding sites [1]. The actions of these proteins can also lead to nucleosome movement and changes in chromatin conformation, resulting in profound transcriptional activation (or repression) of a gene or region [1].

Chromodomain-helicase-DNA-binding genes (CHD) encode a novel class of Swi/Snf proteins that not only contain a Swi/Snf-like helicase ATPase domain but also additional functional domains [2], [3]. These proteins have a DNA-binding domain as well as a chromodomain motif that can directly effect chromatin structure and gene transcription. There is increasing evidence that CHD protein complexes can have a profound effect on chromatin structure and gene expression. Therefore, it is likely that they play an important role in regulating development, cell cycle control, and oncogenesis [4]. CHD is a super family that can be subdivided into five subfamilies based on the presence of specific protein motifs, which endow each family of proteins with a unique function. CHD5 is most similar to CHD3 and CHD4 in that it is contains plant homeodomain motifs. CHD5 was recently identified as a novel tumor suppressor that maps to 1p36, which is frequently deleted in many types of human cancers [5], [6], and the chromatin-remodeling activity of CHD5 is required for appropriate transcriptional activation of the p19^Arf^/p53 pathway [7].

It is clear that CHD5 deficiency is a common initiating event in human tumorigenesis. CHD5 is frequently downregulated through promoter hypermethylation in gastric, breast, ovarian, and glioma tumors [8], [9], [10], [11], suggesting epigenetic silencing of CHD5 by methylation may contribute to tumorigenesis in these tissues. Colorectal cancer (CRC) is one of the three most prevalent cancers in the United States [12] and CHD5 is frequently hypermethylated in human colon cancer cell lines and primary tumors [2], [13], [14].

Although there are many studies on the methylation status of CHD5 in different types of tumors, there are few studies on how another important epigenetic mechanism, microRNAs (miRNAs), may also play a critical role in CHD5 deficiency during colorectal tumorigenesis. miRNAs are small, non-coding RNA molecules present in animals, plants, and viruses that are primarily involved in gene silencing by imperfect base pairing with the 3′-untranslated regions (3′-UTR) of specific mRNAs, which induces mRNA degradation [15]. The loose binding constraints allow one miRNA to bind to several sites within one 3′-UTR and to multiple mRNA targets within the transcriptome, endowing miRNAs with the ability to inhibit several genes at once [16]. Many miRNAs are conserved across widely diverse phyla, indicating their physiological importance [15]. miRNAs play a key role in regulating diverse cellular processes, including development, differentiation, cell growth, apoptosis, viral infection, and metabolism [17].

Some of the miRNAs that are dysregulated in cancer function as tumor suppressors or oncogenes [18]. Several such miRNAs have been identified in colorectal cancer, including the upregulated miR-31, miR-96, miR-135b, and miR-183 and the downregulated miR-133b and miR-145 [19]. Since miRNAs bind the 3′-UTR of their target mRNAs by base pairing, the region of complementarity between a miRNA and its mRNA target is small. This region encompasses nucleotides 2–7 from the 5′-end of the miRNA and is referred to as the ‘seed’ region [20]. Different computational approaches have been developed to predict miRNA target sites throughout the genome [21]. By using the informatic tools MiRanda, PicTar, and TargetScan, miR-211 was predicted to base pair with the 3′-UTR of CHD5, but *in vitro* and *in vivo* experiments are necessary to confirm whether miR-211 actually targets CHD5.

In the present study, we investigated the role of miR-211 on colorectal cancer cells through its downregulation of CHD5 *in vitro* and *in vivo*. First, we examined the expression levels of miR-211 and CHD5 in the colorectal cancer cell lines RKO and HCT-116. We then selected HCT-116 and established the miR-211-stably transfected cell line HCT-116^miR-211^. Finally, we used this cell line to perform a series of *in vitro* and *in vivo* experiments in order to determine whether miR-211 targets CHD5.

## Results

### Expression levels of CHD5 protein and miR-211 are inversely correlated in human colon cancer cell lines

**Figure 1:**
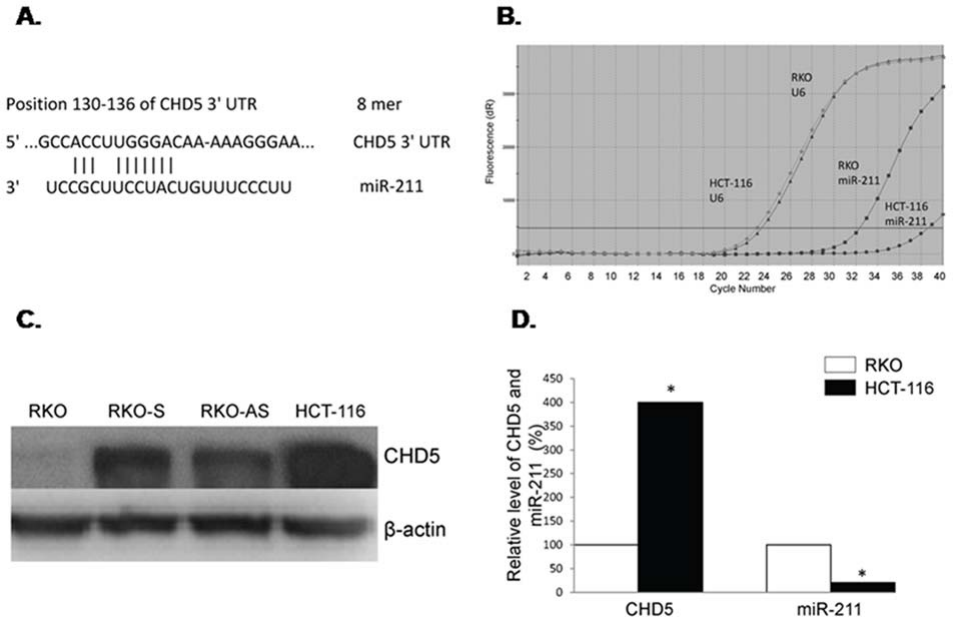
Comparison of CHD5 and miR-211 expression in various colorectal cancer cell lines. (A) RNA sequence map of the 3′-UTR of CHD5 (Gene ID 26038) mRNA with the complementary site (3′-UTR 130–136) for the seed region of miRNA-211. (B) miR-211 levels in RKO, HCT-116, RKO-S, and RKO-AS cell lines by quantitative RT-PCR based on miR-211/U6 expression fold values. (C) CHD5 protein levels in two colon cancer cell lines (RKO and HCT-116) and two established cell lines (RKO-S and RKO-AS) with enforced CHD5-S expression were analyzed by Western blot and semi-quantified based on CHD5/β-actin relative intensities. Bio-Rad Quantity One software was used for densitometric analysis of the Western blots. (D) Comparison of CHD5 and miR-211 expression levels in HCT-116 and RKO cell lines. * indicated as P<0.05.

miR-211 was chosen as a candidate miRNA for targeting CHD5 due to its imperfect base pairing to the 3′-UTR of CHD5 (Fig. 1A). We investigated the expression level of miR-211 in these two cell lines by quantitative real-time RT-PCR (Fig. 1B). In order to select the appropriate cell lines for our study, we evaluated the level of CHD5 protein in four colon cancer cell lines by Western blot analysis (Fig. 1C), which included two previously established cell lines (RKO and HCT-116) and two CHD5 transfected cell lines (RKO-S and RKO-AS). Our results revealed that HCT-116 had the highest expression of CHD5 and RKO had the lowest expression of CHD5. Therefore, HCT-116 and RKO cells lines were used for subsequent experiments. We found an inverse correlation of CHD5 and miR-211 expression (Fig. 1D). The expression level of CHD5 in HCT-116 was 4-fold higher than in RKO, while the expression level of miR-211 in HCT-116 was only 20% of that in RKO. Since HCT-116 cells had both the highest expression level of CHD5 and lowest expression level of miR-211, this cell line was chosen as a candidate cell line to stably transfect with miR-211 for further investigation into the function of miR-211 in the regulation of CHD5 expression under cell culture and tumor xenograft conditions.

### Stably enforced expression of miR-211 directly downregulates CHD5 protein levels in HCT-116 cells

**Figure 2:**
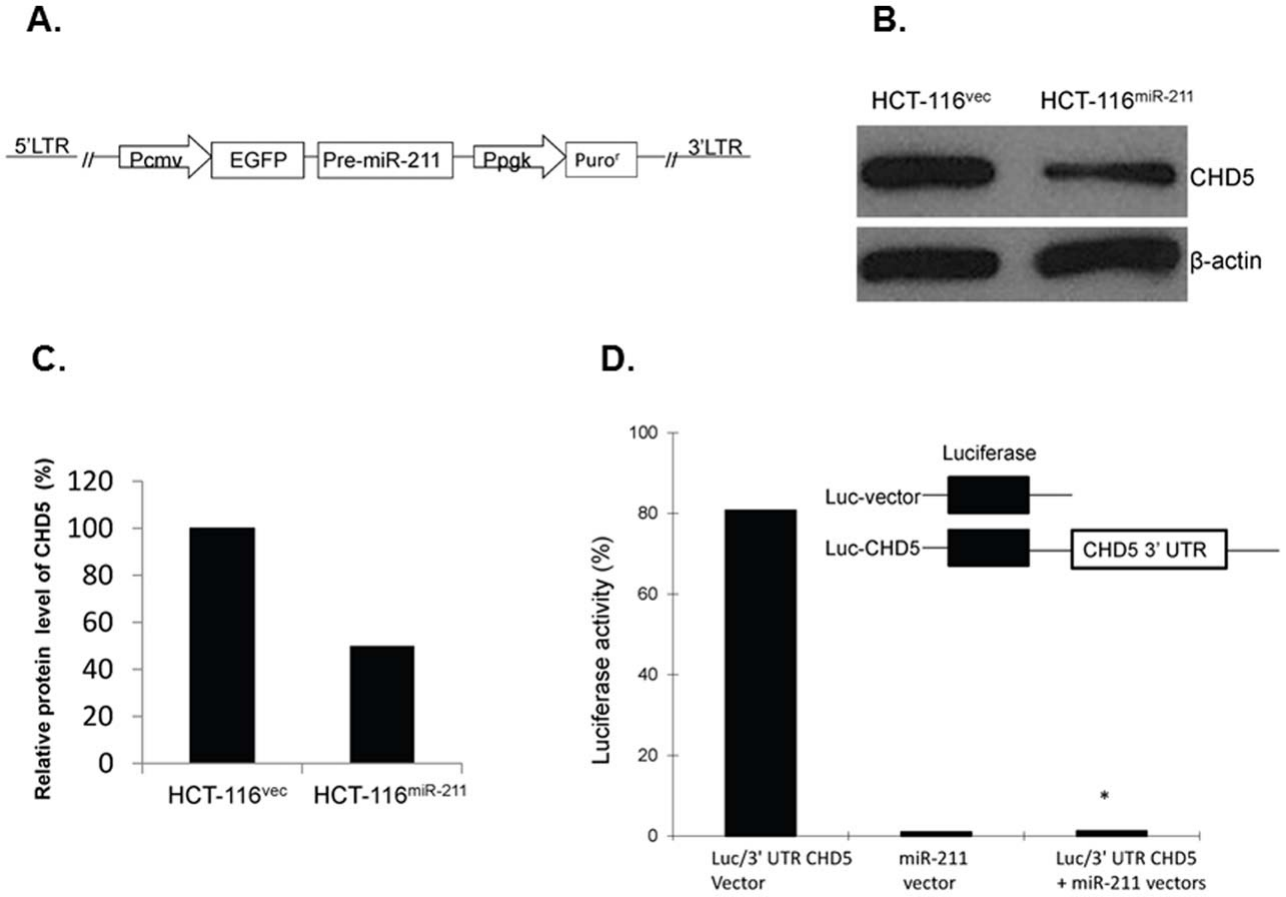
miR-211 inhibits CHD5 expression in colorectal cancer cells stably expressing exogenous miR-211. (A) Schematic representation of the miR-211 vector, which contains an expression cassette of the P_CMV_ promoter, EGFP, and miR-211 precursor and a selective cassette of the P_PGK_ promoter and Puro^r^. (B) CHD5 protein levels in cell lines HCT-116^vec^ and HCT-116^miR-211^ were evaluated by Western blot and (C) semi-quantified based on CHD5/β-actin relative intensities. (D) Luciferase reporter assay using HEK 293T cells that were transfected with either the luciferase/3′-UTR miR-211 reporter vector, the miR-211 vector, or both. Schematic representation of the luciferase-CHD5 reporter vector and luciferase reporter control vector were also inserted. * indicated as P<0.05.

In order to investigate the role of miR-211 in the downregulation of CHD5 under cell culture conditions, we constructed a HCT-116 cell line that stably expressed miR-211 using a lentiviral-delivery system to insert an expression cassette with a P_CMV_ promoter, EGFP, and miR-211 precursor (Fig. 2A). We also constructed a HCT-116 cell line, HCT-116^vec^, that stably expressed the GFP control vector. Fourteen days after lentiviral infection, nearly all HCT-116 cells had detectable levels of green fluorescence, which is an indicator of efficient infection. The lentiviral-delivered EGFP-miR-211 expression was quantified by real-time RT-PCR. miR-211 expression in HCT-116^miR-211^ increased by 16-fold (*p*<0.05). In addition, the expression level of CHD5 decreased significantly (Fig. 2B), with the protein level of CHD5 in HCT-116^miR-211^ cell at only 50% of that in HCT-116^vec^ cells (Fig. 2C). In addition, CHD5 was confirmed as a direct target of miR-211 by a luciferase reporter assay. Fluorescence activity decreased over 90% after 293T cells were co-transfected with the miR-211 expression vector and 3′-UTR of CHD5 mRNA luciferase reporter vector compared to the 3′-UTR of CHD5 luciferase reporter vector alone (Fig. 2D).

### Enforced miR-211 expression increases the proliferation and migration of colon cancer cells *in vitro*

**Figure 3:**
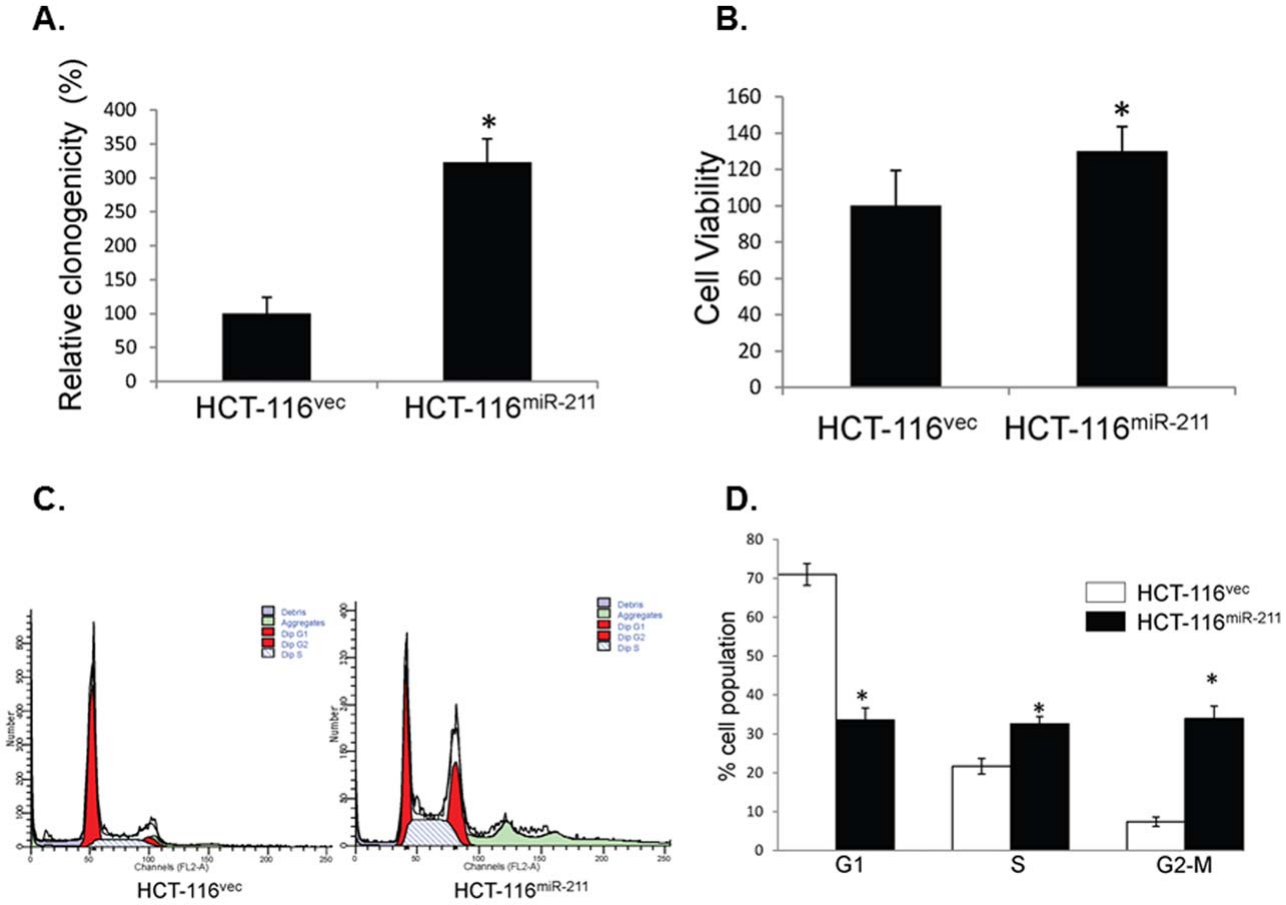
Exogenous miR-211 potentiates proliferation and growth of colorectal cancer cells *in vitro*. Cell proliferation and cell viability of cultured HCT-116^vec^ and HCT-116^miR-211^ cell lines were compared using (A) colony formation assay, (B) MTT assay,and (C and D) cell cycle profiles and the distribution of cells in G1, S, and G2-M phases for each group were analyzed by flow cytometry. The results represent the mean ± SD of two independent experiments with triplicates and * indicated as P<0.05.

**Figure 4:**
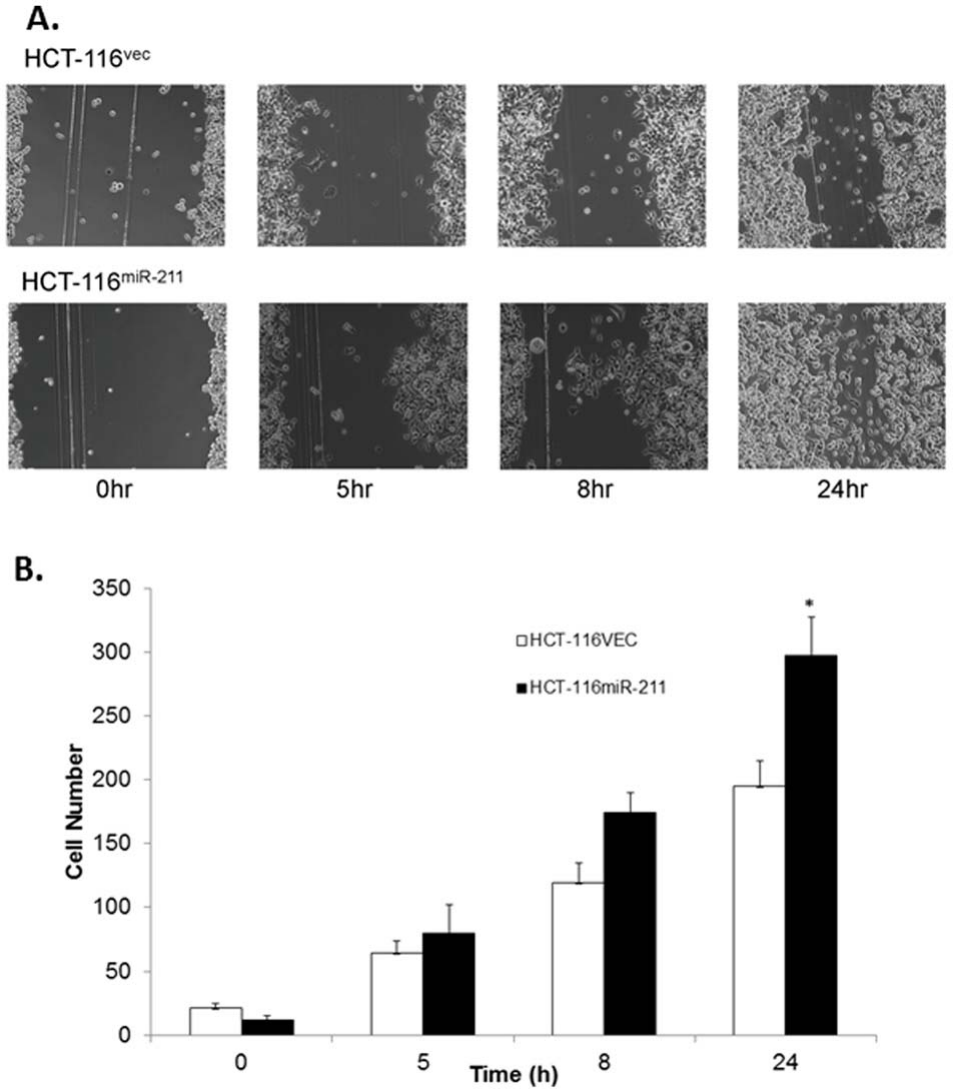
Effects of exogenous miR-211 on the migration ability of colorectal cancer cell lines. (A) The scratched open areas were observed at hours 0, 5, 8, and 24 for HCT-116^vec^ and HCT-116^miR-211^ cell lines under a light microscope at ×400 magnification. (B) The cells were counted from the various time period in the same size of scratch area as referenced at time 0 hr. The results of the scratch assay were from two independent experiments with triplicates and * indicated as P<0.05.

The effect of miR-211 on cell proliferation was assessed using a colorimetric formation assay and cell viability was measured using the MTT assay. HCT-116^vec^ and HCT-116^miR-211^ cells were seeded in separate 6-well plates and cell growth was monitored daily under a microscope for ten days until the colonies were clearly visible to the naked eye. As expected, HCT-116^miR-211^ cells displayed significantly increased proliferation, leading to the formation of more colonies. The colony forming ability of HCT-116^miR-211^ cells was 221% (*p*<0.05) more than that of HCT-116^vec^ cells (Fig. 3A). The MTT assay showed that enforced expression of miR-211 in HCT-116^miR-211^ cells led to a 30% increase in cell proliferation compared to HCT-116^vec^ cells (Fig. 3B). The cell cycle measurements by FCM showed that the proportion of cells in G1 phase decreased by 53% (*p*<0.05), and that in S phase increased by 51% (*p*<0.05) in HCT-116^miR-211^ cells, respectively, compared to HCT-116^vec^ cells (Fig. 3C, D). We also tested the effect of miR-211 on cell migration by scratch assay (Fig. 4A). We found that enforced miR-211 expression significantly increased the potential of HCT-116 cells to migrate when compared to the control cells. The HCT-116^miR-211^ cells were notably migrated into the scratch area at the reference time 5 hr, and the cells were almost crossed over the scratch area at the time 24 hr (Fig. 4A). There was approximately 42.2% more cells in the scratch area of HCT-116^miR-211^ cell plate than HCT-116^vec^ cells (Fig. 4B).

### Enforced miR-211 increases the proliferation of colon cancer cells *in vivo*

**Figure 5:**
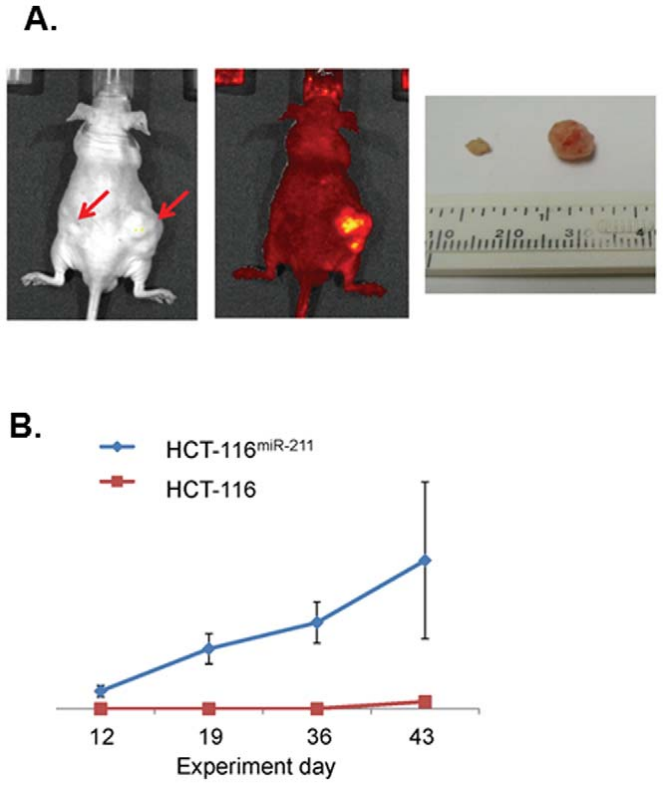
Exogenous miR-211 potentiates proliferation and growth of colorectal cancer cells *in vivo*. (A) Tumor xenograft growth of HCT-116 and HCT-116^miR-211^ are shown under a light and fluorescence imaging system. The tumor mass (g) was measured on the final experimental day immediately after the tumor tissue was removed from the mouse by surgical excision. Tumor xenograft growth for HCT-116 and HCT-116^miR-211^ groups was compared. (B) The day of cell inoculation was the experimental start day and all mice were sacrificed on day 43. The growth of solid tumor xenografts was monitored once a week and measured using vernier calipers.

In addition to examining the biological functions of miR-211 *in vitro*, we also assessed the *in vivo* function of miR-211 using a xenograft transplantation model. By subcutaneously transplanting the HCT-116^vec^ or HCT-116^miR-211^ cells into nude mice, we monitored tumor growth over a 6-week period. As shown in Figure 5, after 6 weeks a solid tumor had formed in the right flank of each of the mice with the average size of 284 mm^3^ and an average weight of 0.592 g. However, much smaller tumors were formed in the left flank with an average size of 13.55 mm^3^ and an average weight of 0.028 g, suggesting that miR-211 increases the proliferation of colon cancer cells *in vivo*.

### Enforced expression of miR-211 alters the expression of growth-associated proteins and p53-related pathway proteins

**Figure 6:**
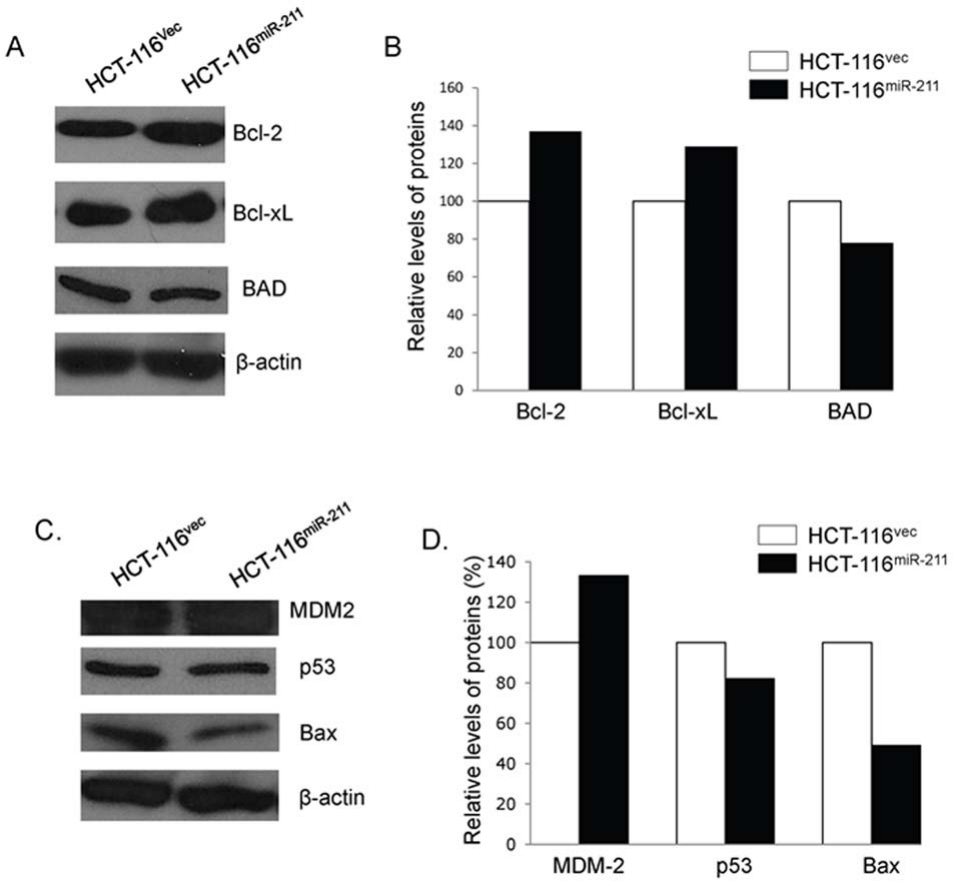
Effects of exogenous miR-211 on cell growth-associated proteins and p53-related pathway proteins in colorectal cancer cell lines. (A) The levels of cell growth-associated proteins in HCT-116^vec^ and HCT-116^miR-211^ cell lines were analyzed by Western blot and (B) semi-quantified based on targeted protein/β-actin relative intensities. The levels of p53-related pathway proteins in HCT-116^vec^ and HCT-116^miR-211^ cell lines were analyzed by Western blot (C) and semi-quantified based on targeted protein/β-actin relative intensities (D).

The expression levels of the cell survival-supporting proteins Bcl-2 and Bcl-xL in HCT-116^miR-211^ were increased by 37% and 29%, respectively, while the expression level of the death-promoting protein Bad was decreased by 22% (Fig. 6A, B). Expression of Mdm-2, a negative regulator of p53, was increased 33% in HCT-116^miR-211^ cells compared to HCT-116^vec^ cells. However, expression of p53 and its target gene Bax decreased by 18% and 51%, respectively (Fig. 6C, D).

## Discussion

CHD5 is a newly identified tumor suppressor gene [2] located at 1p36 [7]. Its reduced expression occurs in many tumor types due to hypermethylation of its promoter [8], [9], [11]. Several studies have suggested that CHD5 positively regulates p53-mediated pathways. The downregulation and hypermethylation of CHD5 has been found in CRC [2], [13], [15]. However, little is known regarding whether miRNAs, another aspect of epigenetic regulation, impact CHD5 expression and the phenotype of CRC cells.

MiRNAs are predicted to regulate over 30% of all gene expression and may account for some of the aberrant gene expression in cancer cells. We used bioinformatic tools and found that miR-211 is predicted to base pair to the 3′-untranslated region (3′-UTR) of CHD5 (Fig. 2A). miR-211 is significantly upregulated in other types of cancer [22] and may function as an oncogene, which is consistent with the tumor suppressor function of CHD5. However, whether miR-211 directly targets CHD5 and affects the phenotype of cells from the intestine needed to be confirmed. In the present study, we determined that enforced expression of miR-211 in a colorectal cancer cell line directly downregulates CHD5, resulting in increased cell viability, cell cycle progression, and migration ability and decreased apoptosis. We confirmed our results at several different levels, including cell culture, tumor xenografts, and protein changes in CHD5-related pathways.

We successfully established a colon cancer cell line that stably expressed EGFP-miR-211, HCT-116^miR-211^, using stable gene transfection technology (Fig. 2A). RKO and HCT-116 are both colorectal cancer cell lines. CHD5 was lowly expressed in RKO and highly expressed in HCT-116 cells [14]. The expression levels of CHD5 and miR-211 in RKO, RKO-S, RKO-AS, and HCT-116 were detected by Western blot and real-time PCR. While RKO-S is CHD5-stably transfected cell line, HCT-116 had the highest expression level of CHD5. Since HCT-116 also had the lowest expression level of miR-211, we selected this cell line to stably transfect with miR-211 (Fig. 1D). We successfully established enforced EGFP-miR-211 expression in the colon cell line HCT-116^miR-211^ using the Lenti-X™ Lentiviral Expression System. We also established a cell line transfected with the empty EGFP vector, HCT-116^vec^. Since the EGFP gene shared the same P_CMV_ promoter with the miR-211 gene, we were able to easily monitor miR-211 expression in the cells under a fluorescent microscope. Since the expression of miR-211 in HCT-116 and HCT-116^vec^ cells was similar, HCT-116^vec^ was used as the control cell line for the following experiments. The expression level of miR-211 was 15-fold higher in HCT-116^miR-211^ cells compared to HCT-116^vec^cells.

In addition, the level of CHD5 expression was significantly reduced in HCT-116^miR-211^ cells compared with HCT-116^vec^ cells (Fig. 2B, C). Enforced expression of miR-211 in HCT-116 cells increased proliferation, colony formation, viability (Fig. 3), and migration ability (Fig. 4) *in vitro*. Cell transit from G0/G1 to S phase was accelerated (Fig. 3C,D) and expression of cell survival-supporting proteins Bcl-2 and Bcl-xL increased while expression of the death-promoting protein Bad decreased (Fig. 6A,B). Upregulation of miR-211 enhanced the proliferation of colorectal cancer cells may be by downregulating CHD5 (Fig. 2D) and also promoted tumor growth *in vivo*. Six weeks after subcutaneous transplantation of HCT-116^miR-211^ and HCT-116 cells into nude mice, HCT-116^miR-211^ developed into tumors with an average weight of 0.592 g while HCT-116 cells only formed much smaller tumors with an average weight of 0.028 g. Taking into accounts that HCT-116 and HCT-116^vec^ didn't show any significant differences in biological behavior, our results reveal that miR-211 promotes tumor growth of xenografts *in vivo* (Fig. 5).

Interestingly, downregulation of CHD5 by miR-211 impacts the phenotype of colorectal cell line by affecting the expression of p53-related pathway proteins (Fig. 6C, D). p53 is one of the most studied tumor suppressors and over 50% of human tumors carry loss of function mutations [23]. p53 suppresses tumorigenesis through the induction of cell-cycle-arrest or apoptosis programs in response to a plethora of different cellular stress signals [24]. The tumor suppressor function of p53 also includes other mechanisms, including regulating metabolic pathways [25]. CHD5 appears to modulate carcinogenesis through a p53-related pathway [7]. In our study, we found that upregulation of miR-211 in HCT-116 cells resulted in downregulation of CHD5, affecting the expression of several p53-regulated pathway proteins, including Mdm-2, p53, and Bax. The level of Mdm-2, a negative regulator of p53, was increased while the levels of p53 and its target gene, Bax, were decreased, indicating that CHD5 deficiency compromises p53-regulated pathways and facilitates tumorigenesis. The malignant phenotype of the HCT-116 cells was increased by upregulation of miR-211 and the enhancement of proliferation and migration of HCT-116 cells may be involved subsequent alterations in p53-related pathways. Further studies will be needed to determine whether p53-related signaling is the sole target for miRNA-211 regulation of CHD5.

Taken together, the aberrant expression of miR-211 and subsequent change in the level of CHD5 are associated with colorectal tumorigenesis. Our results demonstrate that enforced expression of miR-211 in the colorectal cancer cell line HCT-116 increased the malignant phenotype of the cells by directly downregulating the expression of CHD5 and may be by modulating p53-related pathways. Specifically, enforced expression of miR-211 enhanced promotion and migration, reduced apoptosis, accelerated the transition from G0/G1 to S phase *in vitro* and enhanced proliferation *in vivo*. However, since one single miRNA can modulate several different targets the other possible roles of miR-211 in colorectal cancer need further study. In addition, more studies are needed to validate the relationship between miR-211 and CHD5 in human colorectal cancer development and prognosis. Our study provides a better understanding of regulatory capacity of the miR-211 in promoting colorectal tumorigenesis by targeting CHD5 expression.

## Materials and Methods

### Cell lines and culture

Human colon cancer cell lines (RKO and HCT-116) and a human embryonic kidney cell line (HEK293T) were obtained from the American Type Culture Collection (ATCC, Manassas, VA, USA). We used two CHD5-Sens and CHD5-antiSens forced expression stably established RKO cell lines (CHD5 upregulated cell line RKO-S and its anti-sense RKO-AS) [26]. RKO cells were cultured in MEM and RKO-S and RKO-AS cells were cultured in MEM containing 900 µg/ml G418. HCT-116 cells were cultured in McCoy's 5A medium (ATCC). HEK293T cells were cultured in Dulbecco's Modified Eagle's medium (DMEM; Gibco, USA). All media was supplemented with 10% fetal bovine serum and 1% Penicillin/Streptomycin, and all cell lines were maintained at 37°C in 5% CO_2_ and 95% humidity.

### Identification of microRNA targets

The algorithms of MiRanda (http://www.sanger.ac.uk), PicTar (http://pictar.bio.nyu.edu), and TargetScan (http://www.targetscan.org/) were used to predict which human miRNAs may bind to the 3′-UTR of CHD5 (Gene ID 26038). Briefly, these algorithms provide 3′-UTR alignments with predicted sites and links to various public databases for the prediction of miRNA binding sites.

### miRNA extraction and quantitative RT-PCR

Total RNA was extracted using the RNeasy Mini Kit (Qiagen, Germantown, MD, USA). A miR-211 forward primer (5′-TTCCCTTTGTCATCCTTCGCCT-3′) was synthesized at Sigma (Woodlands, TX). Total RNA was polyadenylated by polyA polymerase from *E. coli*, and then first-strand cDNA synthesis and quantitative PCR were conducted with the High-Specificity miRNA QRT-PCR Detection kit (Stratagene, La Jolla, CA). Polymerase chain reaction (PCR) was performed on a Mx3000P™ Instrument (Stratagene Laboratory), with U6 as an internal control because of its stable expression across human tissues and cell lines, as suggested by the manufacturer and other investigators [27]. We used the comparative threshold cycle (Ct) method to measure relative changes in miRNA expression. Ct is the cycle number at which the fluorescence signal of the amplification plot passes a fixed threshold. ΔCt = Ct(miR-211)−Ct(U6), ΔΔCt = ΔCt(vector)−ΔCt (miR-211). Expression fold value = 2^−ΔΔCt^. All experiments were done in triplicate. A negative control without a template was run in parallel to assess the overall specificity of the reaction.

### Stable transfection of miR-211

We modified the commercial pLVX-Tight-Puro vector (Clontech, Mountain View, CA) with an expression cassette containing the P_CMV_ promoter, EGFP, miRNA linker, and pre-miR-211 by replacing the P_tight_ promoter [28]. The miRNA linker contained a multiple cloning site. The pre-miR-211 double strand sequence was designed based on miRBase Sequence database version 12.0. The constructed vectors were verified by DNA sequencing. The recombinant lentiviral particles containing either the miR-211 vector or the EGFP control vector were transfected into HEK 293T packaging cells using the lentiphos™ HT packaging system and following the manufacturer's instructions (Clontech, Mountain View, CA). HCT-116 cells were grown to 70–80% confluence in 6-well plates and infected with 200 µl of lentivirus containing either the miR-211 vector or the control vector for 2 h. Afterwards, 2 ml of McCoy's 5A media with 10% FBS was added to each well. After 48 h, the infected cells were selected with fresh medium containing 5 µg/ml puromycin for 4–5 passages. Cell lines stably expressing either miR-211 or the control vector were generated and the infected cells could be viewed under a fluorescence microscope.

### Luciferase reporter assay

A luciferase reporter assay was used to verify that the complementary sequence of miR-211 binds to the 3′-UTR of CHD5 mRNA. A fragment of CHD5 mRNA, which included miR-211's binding site was cloned into the phCMV-FSR luciferase reporter vector (Genlantis, San Diego, CA). HEK 293T cells were seeded in a 24-well plate so that they were 50% confluent at the time of transfection. The luciferase reporter vector and either the GFP control vector or miR-211 expression vector were cotransfected in HEK 293T cells using calcium phosphate precipitation. Luciferase activity was measured in live cells 48 h later according to the manufacturer's protocol using bioluminescence imaging with a Xenogren IVIS instrument (Caliper Life Sciences, Hopkinton, MA).

### 3-(4, 5-dimethylthiazol-2-yl) 2, 5-diphenyltetrazolium bromide (MTT) assay

An MTT assay was performed as previously described [29]. Briefly, cells were seeded into 96 well plates at an initial density of 5,000 cells/well. After 72 h, 200 µl of MTT solution (5 mg/mL, Sigma, St Louis, MO) was added to each well and incubated for 4 h at 37°C. The supernatant was then discarded, and 200 µl of dimethyl sulfoxide (DMSO) was added to each well to dissolve the precipitate. After 30 min at room temperature the plates were scanned spectrophotometrically with a microplate reader (Bio-Rad, Hercules, CA) set at 560 nm to measure the absorbance. Each test was repeated in five wells.

### Colony formation assay

A colony formation assay was performed as previously described [29]. Briefly, HCT-116^vec^ and HCT-116^miR-211^ cells were plated into 6-well tissue culture plates (Palo Alto, CA). Five hundred cells of HCT-116^vec^ and HCT-116^miR-211^ were seeded into each well in triplicate, incubated for 10 days, and colonies were stained with 0.1% trypan blue in 50% ethanol. Colonies containing 50 or more cells were counted as viable clonogenic cells. At least two independent experiments were performed.

### Cell cycle assay

Cell cycle analysis was performed as previously described [29]. Briefly, HCT-116^vec^ and HCT-116^miR-211^ cells in the log phase of growth were collected and fixed with cold 75% ethanol and stored at −20°C for 24 h. After the ethanol was removed, the cells were incubated with 1 mg/ml RNase A in PBS for 30 min at room temperature, and then the cells were incubated an additional 30 min in the dark in 0.5 ml of 50 mg/ml propidium iodide. The distribution of cells throughout the cell cycle was analyzed by Becton-Dickinson FACScan, and DNA histograms were analyzed with modified software. Each test was repeated in triplicate.

### The scratch assay

Briefly, an open area or ‘scratch’ was produced in 90% HCT-116^miR-211^ cell monolayers using a 200 µl pipette tip as previously described [30]. HCT-116^miR-211^ cells were washed with PBS to remove the displaced cells in the open area, and cultured in McCoy's 5A for designated times. HCT-116^vec^ were similarly treated and cultured in McCoy's 5A. Migration into the open area was observed by the naked eye.

### Western blot analysis

HCT-116^vec^ and HCT-116^miR-211^ cells were washed with cold PBS and lysed in RIPA lysis buffer on ice for 30 min. Homogenates of cells were clarified by centrifugation at 16,000 g for 30 min at 4°C and the protein concentration was measured using Quick Start Bradford Dye Reagent (Bio-Rad). Forty µg of protein from each sample was subjected to SDS-PAGE on an SDS-acrylamide gel. The separated proteins were transferred to PVDF membranes and incubated with a primary antibody followed by incubation with a secondary antibody. The specific protein was detected using Bio-Rad equipment (Thermo Spectronic Company). Primary antibodies used for Western blot analysis included rabbit anti-human CHD5 (Abcam; 1∶5,000 dilution), mouse anti-human Bcl-2 (Sigma; 1∶1,000 dilution), rabbit anti-human Bcl-xL (Sigma; 1∶1,000 dilution), rabbit anti-human Bad (Santa Cruz; 1∶500 dilution), rabbit anti-human Bax (Sigma; 1∶500 dilution), rabbit anti-human p53 (Sigma; 1∶1,000 dilution), mouse anti-human MDM-2 (Sigma; 1∶1,000 dilution), and mouse anti-human β-actin (Sigma; 1∶5,000 dilution) and secondary antibodies included goat anti-rabbit IgG-HRP (Santa Cruz; 1∶2,000 dilution) and goat anti-mouse IgG-HRP (Santa Cruz; 1∶2,000 dilution). Mouse anti-human β-actin was used as a control.

### *In vivo* xenograft tumor growth in nude mice

The *in vivo* test was performed as previously described [29]. Four-week-old, male Balb/c athymic nude mice (Nu/Nu) were obtained from Harlan Sprague Dawley, Inc. (Indianapolis, IN). Mice were housed in temperature-controlled rooms (74±2°F) with a 12-hour alternating light-dark cycle. Approximately 3×10^6^ cells in log phase were resuspended in McCoy's 5A medium and injected subcutaneously into the mice. To minimize individual differences, HCT-116 cells and HCT-116^miR-211^ cells were injected into the left and right flanks of each mouse, respectively. The six mice were housed for 6 weeks post inoculation. The mice were euthanized and the tumor tissues were removed by surgical excision. Tumor volume was measured once a week during the experimental period. Guidelines for the humane treatment of animals were followed as approved by the Howard University Animal Care and Use Committee (IACUC MED 10-10 R3).

### Statistical analysis

Data are expressed as the mean ± standard deviation (S.D.). *p*<0.05 was considered statistically significant for ANOVA and STD t tests.

## References

[1] Sudarsanam P, Winston F (2000). The Swi/Snf family nucleosome-remodeling complexes and transcriptional control.. Trends Genet.

[2] Mokarram P, Kumar K, Brim H, Naghibalhossaini F, Saberi-firoozi M (2009). Distinct High-Profile Methylated Genes in Colorectal Cancer.. PLoS One.

[3] Woodage T, Basrai MA, Baxevanis AD, Hieter P, Collins FS (1997). Characterization of the CHD family of proteins.. Proc Natl Acad Sci USA.

[4] Bagchi A, Mills AA (2008). The Quest for the 1p36 Tumor Suppressor.. Cancer Res.

[5] White PS, Thompson PM, Gotoh T, Okawa ER, Igarashi J (2005). Definition and characterization of a region of 1p36.3 consistently deleted in neuroblastoma.. Oncogene.

[6] Aarts M, Dannenberg H, deLeeuw RJ, van Nederveen FH, Verhofstad AA (2006). Microarray-based CGH of sporadic and syndrome-related pheochromocytomas using a 0.1–0.2 Mb bacterial artificial chromosome array spanning chromosome arm 1p.. Genes Chromosomes Cancer.

[7] Bagchi A, Papazoglu C, Wu Y, Capurso D, Brodt M (2007). CHD5 Is a Tumor Suppressor at Human 1p36.. Cell.

[8] Wang X, Lau KK, So LK, Lam YW (2009). CHD5 is down-regulated through promoter hypermethylation in gastric cancer.. J Biomed Sci.

[9] Gorringe KL, Choong DY, Williams LH, Ramakrishna M, Sridhar A (2008). Mutation and Methylation Analysis of the Chromodomain-Helicase-DNA Binding 5 Gene in Ovarian Cancer.. Neoplasia.

[10] Fujita T, Igarashi J, Okawa ER, Gotoh T, Manne J (2008). CHD5, a Tumor Suppressor Gene Deleted From 1p36.31 in Neuroblastomas,. J Natl Cancer Inst.

[11] Tang YY, Tian DA, Yan W, Xia LM, Zhang lQ (2008). Involvement of CHD5 in Genesis and Development of Hepatoma as a Potent Anti-oncogene.. Chin J Gastroenterol Hepatol.

[12] Jemal A, Siegel R, Xu J, Ward E (2010). Cancer Statistics, 2010,. CA Cancer J Clin.

[13] Lin Y, Li YY, Nie YQ, Sha WH (2007). Quantitative Expression of Tumor Suppressor CHD5 in Colorectal Cancer Tissue,. Chin J Dig.

[14] Mulero-Navarro S, Esteller M (2008). Chromatin remodeling factor CHD5 is silenced by promoter CpG island hypermethylation in human cancer.. Epigenetics.

[15] Kanellopoulou C, Monticelli S (2008). A role for microRNAs in the development of the immune system and in the pathogenesis of cancer.. Semin Cancer Biol.

[16] Voorhoeve PM, Agami R (2007). Classifying microRNAs in cancer: The good, the bad and the ugly.. Biochim Biophys Acta.

[17] Scholzová E, Malík R, Sevcík J, Kleibl Z (2007). RNA regulation and cancer development.. Cancer Lett.

[18] Gartel AL, Kandel ES (2008). miRNAs: Little known mediators of oncogenesis.. Semin Cancer Biol.

[19] Kim S, Choi M, Cho KH (2009). Identifying the target mRNAs of microRNAs in colorectal cancer.. Comput Biol Chem.

[20] Lewis BP, Burge CB, Bartel DP (2005). Conserved Seed pairing, often anked by adenosines, indicates that thousands of human genes are MicroRNA targets.. Cell.

[21] Rajewsky N (2006). microRNA target predictions in animals.. Nat Genet.

[22] Chang KW, Liu CJ, Chu TH, Cheng HW, Hung PS (2008). Association between High miR-211 microRNA Expression and the Poor Prognosis of Oral Carcinoma.. J Dent Res.

[23] Ozaki T, Nakagawara A (2011). p53: the attractive tumor suppressor in the cancer research field.. J Biomed Biotechnol.

[24] Brady CA, Attardi LD (2010). p53 at a glance.. J Cell Sci.

[25] Maddocks OD, Vousden KH (2010). Metabolic regulation by p53.. J Mol Med.

[26] Ashktorab H, Schäffer AA, Daremipouran M (2010). Distinct genetic alterations in colorectal cancer.. PLoS One.

[27] Yantiss RK, Goodarzi M, Zhou XK (2009). Clinical, pathologic, and molecular features of early-onset colorectal carcinoma.. Am J Surg Pathol.

[28] Hao Y, Gu X, Zhao Y, Greene S, Sha W (2011). Enforced Expression of miR-101 Inhibits Prostate Cancer Cell Growth by Modulating the COX-2 Pathway In Vivo.. Cancer Prev Res (Phila).

[29] Zhao Y, Hao Y, Ji H, Fang Y, Guo Y (2010). Combination effects of salvianolic acid B with low-dose celecoxib on inhibition of head and neck squamous cell carcinoma growth in vitro and in vivo.. Cancer Prev Res (Phila).

[30] Liang CC, Park AY, Guan JL (2007). In vitro scatch assay: a convenient and inexpensive method for analysis of cell migration in vitro.. Nat Protoc.

